# Urea-Derived Carbon Nitride Fragmentation: A Route to Melem-Based Superstructures

**DOI:** 10.3390/polym18212664

**Published:** 2026-10-28

**Authors:** Milica V. Carević, Nadica D. Abazović, Zorica D. Mojović, Jugoslav B. Krstić, Miloš D. Mojović, Tatjana D. Vulić, Mirjana I. Čomor

**Affiliations:** 1 Vinča Institute of Nuclear Sciences, National Institute of the Republic of Serbia, University of Belgrade, Mike Petrovića Alasa 12–14, Vinča, 11351 Belgrade, Serbia; tanja030@vin.bg.ac.rs (T.D.V.); mirjanac@vin.bg.ac.rs (M.I.Č.); 2 University of Belgrade, Institute of Chemistry, Technology and Metallurgy, Department of Catalysis and Chemical Engineering, Njegoševa 12, 11000 Belgrade, Serbia; zorica.mojovic@ihtm.bg.ac.rs (Z.D.M.); jugoslav.krstic@ihtm.bg.ac.rs (J.B.K.); 3 Center for Physical Chemistry of Biological Systems, BioScope Labs, Faculty of Physical Chemistry, University of Belgrade, Studentski trg 12–16, 11158 Belgrade, Serbia; milos@ffh.bg.ac.rs

**Keywords:** graphitic carbon nitride, melem hydrate, interfacial charge transfer, oxidative treatment, X-ray photoelectron spectroscopy, electron paramagnetic resonance

## Abstract

Urea-derived graphitic carbon nitride (g-C_3_N_4_) underwent chemical treatment in a strongly oxidizing H_2_SO_4_/HNO_3_/H_2_O_2_ environment, followed by dialysis against water. XRD and SEM measurements revealed significant disruption of the layered polymeric framework, including loss of hydrogen-bond-mediated order, partial depolymerization into melem-based units, and the formation of rod-like melem hydrate superstructures. FTIR spectra indicated that chemical treatment resulted in the loss of the heptazine structure and the emergence of melem/triazine vibrations. Consistent disruption of the C–N framework was also observed in XPS spectra, which showed a reduced contribution of nitrogen species associated with inter-triazine bridging. Mott–Schottky analysis yielded potential values suggesting the presence of additional electronic states in the chemically treated sample. This aligns with EPR spin-trapping results, which showed approximately a 2.5-fold decrease in superoxide radical anion generation upon visible light irradiation compared with the parent compound, attributed to both reduced visible-light absorption and trapping of photogenerated electrons in defect states.

## 1. Introduction

Graphitic carbon nitride (g-C_3_N_4_) is a conjugated polymeric semiconductor renowned for its excellent photocatalytic properties and has been extensively studied over the past few decades [1]. Depending on the desired final characteristics of the synthesized material, various organic carbon- and nitrogen-rich precursors, such as melamine, cyanamide, dicyanamide, or urea, have been used to obtain g-C_3_N_4_ through simple thermal treatment. The resulting bulk materials are characterized by band gap energy of about 2.7 eV [2], with the conduction band potential at approximately −1.3 eV vs. NHE [2], making them photoactive under visible light irradiation with a sufficiently high overpotential for the production of superoxide anion radicals.

However, regardless of the precursor or synthetic procedure used to obtain it, g-C_3_N_4_ suffers from several intrinsic drawbacks that hinder its photocatalytic performance: rapid recombination of photogenerated charges, a low density of available reactive sites, and poor charge carrier mobility [3]. The origin of these drawbacks lies in the structural arrangement of s-triazine-based melon chains interconnected by covalent and hydrogen bonds, forming layers. These layers are connected by weak van der Waals forces, resulting in a stacked configuration with a substantial interlayer potential barrier—estimated at 33.2 eV—which obstructs electrical transport across layers [4]. Additionally, intralayer electrical transport in hydrogen-bond-rich areas is impeded by a potential barrier of 7.9 eV, causing localization of photogenerated charges and making them unavailable for useful redox reactions [4]. In this context, exfoliating the bulk material into thinner layered structures would enhance electrical transport, simultaneously prolonging the lifetime of photogenerated charges and increasing the availability of active sites, ultimately improving photocatalytic performance [5].

Previously, we have shown that thermally exfoliated g-C_3_N_4_ exhibits improved photocatalytic activity under simulated solar irradiation [6]. In this study, we examine the effect of the chemical exfoliation process in a strongly oxidizing environment on the structural and optical characteristics of urea-derived g-C_3_N_4_. Several studies [2,5,7] have shown that treatment of carbon nitride with concentrated nitric acid leads to its depolymerization. Specifically, protons intercalate between carbon nitride layers, attaching selectively to bridging or stranding nitrogen atoms [2]. Such a swollen polymer matrix, when exposed to strong oxidative agents such as HNO_3_, is prone to depolymerization, that is, decomposition into smaller building blocks [2]. The same effect is reported for carbon nitride (C_3_N_4_ or C_3_N_5_) treated with concentrated sulfuric acid or a combination of concentrated HNO_3_ and H_2_SO_4_: after initial intercalation/protonation and chemical exfoliation, cleavage of bridging C-NH-C bonds between melon units begins, eventually leading to decomposition of carbon nitride into melem units [8,9,10,11,12].

However, no significant changes in structure or morphology, nor fragmentation into smaller pieces, are reported when acid-etched carbon nitride is subsequently treated with a strong oxidizing agent such as hydrogen peroxide. As Choudhury et al. [13] reported, the layered nanostructure of g-C_3_N_4_ was preserved. Nevertheless, this combined chemical treatment resulted in surface functionalization with oxygen-containing groups (carbonyl, carboxyl, hydroxyl, etc.). Similar findings are reported by Kumar et al. [3] and Bahaguna et al. [14].

In this work, we investigate the effect of strongly oxidizing H_2_SO_4_/HNO_3_/H_2_O_2_ treatment on the structure and electronic properties of urea-derived g-C_3_N_4_. While acid-induced depolymerization of carbon nitride has been previously reported, the main contribution of this study is the demonstration that a chemical procedure previously employed for surface functionalization can induce extensive fragmentation of porous urea-derived carbon nitride, followed by the formation of melem hydrate-based superstructures. The accompanying changes in surface chemistry and electronic properties are also examined.

## 2. Materials and Methods

### 2.1. Materials

The following commercial chemicals were used: urea (VWR, reagent grade, Leuven, Belgium), nitric acid (HNO_3_, J.T. Baker, Deventer, The Netherlands, 69–70%), sulfuric acid (H_2_SO_4_, J.T. Baker, Avantor, Radnor, PA, USA, 95–97%), and hydrogen peroxide (H_2_O_2_, Chemsolute, The Geyer, Renningen, Germany, 30%). All reagents were used as received without further purification. Deionized water obtained from a Milli-Q purification system was used in all experiments.

### 2.2. Sample Preparation

**Graphitic carbon nitride (GCN)** was prepared by the thermal polymerization of urea, following the procedure described in our previous work [6]. Briefly, urea (5 g) was heated in a semi-covered crucible from room temperature to 550 °C at a rate of 5 °C min^−1^, after which this temperature was maintained for an additional 2 h. After cooling to ambient temperature, the resulting yellow powder was ground into a fine powder and stored for later use.

**Acid-treated carbon nitride (ACN)** samples were synthesized using a slightly modified procedure reported in the literature [14]. The protocol was as follows: 0.3 g of as-prepared GCN was dispersed in 9 mL of a mixed acid solution composed of concentrated H_2_SO_4_ and HNO_3_ in a 1:1 volume ratio. The resulting suspension was stirred for 30 min. Subsequently, 4 mL of H_2_O_2_ was added dropwise under continuous stirring, and the mixture was stirred for a further 15 min. Appropriate precautions should be taken due to the vigorous nature of the reaction and the release of nitrogen oxide fumes upon addition of H_2_O_2_. After this, 50 mL of deionized water was added to dilute and quench the reaction. The reaction mixture was then left to stand for approximately 1 h to allow sedimentation. All aforementioned steps were carried out at room temperature. The supernatant was carefully removed by pipetting, and 150 mL of deionized water was added. The suspension was transferred into dialysis tubing and dialyzed until the pH of the surrounding water reached neutrality. After dialysis, the product was collected by centrifugation and dried for further use.

### 2.3. Characterization

X-ray diffraction (XRD) patterns were recorded using a Philips PW 1050 powder diffractometer with Ni-filtered Cu Kα radiation (λ = 0.1542 nm). Data were collected in step-scan mode with a step size of 0.05° and a counting time of 5 s per step. Fourier transform infrared (FTIR) spectra were obtained with a Nicolet 380 spectrometer (Thermo Fisher Scientific, Waltham, MA, USA) equipped with a Smart Orbit™ ATR module. Measurements were performed in the 4000–525 cm^−1^ region. A Scios 2 DualBeam (SEM) (Thermo Fisher Scientific, Waltham, MA, USA), equipped with an energy-dispersive X-ray spectrometer, was used to analyze the morphology and qualitative chemical composition of the samples. Measurements were conducted at an accelerating voltage of 10 kV. UV–Vis diffuse reflectance (DR) spectra were collected in the 200–900 nm wavelength range using a Shimadzu UV-2600 spectrophotometer (Shimadzu Corporation, Kyoto, Japan) equipped with an integrating sphere (ISR-2600 Plus for UV-2600, Shimadzu Corporation, Kyoto, Japan). XPS measurements were performed using a SPECS system equipped with an XP50M X-ray source for the Focus 500 and a PHOIBOS 100/150 analyzer (Specs system, Berlin, Germany). An Al Kα radiation source (hν = 1486.74 eV) operated at 12.5 kV and 32 mA was employed. Survey spectra in the 0–1000 eV binding energy range were acquired at a base pressure of 8 × 10^−9^ mbar, using a pass energy of 40 eV in FAT mode. All binding energies were calibrated with respect to the C 1s peak at 284.5 eV. Data were recorded using the SpecsLab (version 2.79, SPECS Surface Nano Analysis, GmbH, Berlin Germany) acquisition software and subsequently processed with the CasaXPS software package (version 2.3., Casa Software Ltd., UK) EPR spectra were recorded at room temperature using a Bruker Elexsys E540 spectrometer (Bruker BioSpin, Rheinstetten, Germany) operating at X-band (9.85 GHz). The measurement parameters were: modulation amplitude, 2 G; modulation frequency, 100 kHz; microwave power, 10 mW; and scan range, 200 G. Data acquisition and processing were carried out using the Xepr software package (Xepr-2.6b.170, Bruker BioSpin, Rheinstetten, Germany). Samples (1 mg) were used either as dry powder or, for spin-trapping experiments, suspended in 26 µL of water containing 4 µL of the spin-trap DEPMPO (final concentration 3.3 mM). The samples were loaded into 5 cm long Teflon tubes (wall thickness 0.025 mm, inner diameter 0.6 mm; Zeus Industries, Raritan, NJ, USA), which were then inserted into quartz capillaries for EPR measurements. For visible-light-induced radical generation, samples were irradiated using an 800 W artificial sunlight LED light source.

Both samples were irradiated outside the cavity under identical conditions (distance from the light source and irradiation time) and then returned to the same position in the cavity, where their spin-trapping spectra were recorded with the same instrument settings. The DEPMPO/OOH signals were compared on the basis of the heights of several characteristic peaks of the experimental spectra.

Electrochemical experiments were conducted using an Autolab electrochemical workstation (Autolab PGSTAT302N, Metrohm-Autolab BV, Utrecht, The Netherlands) and a three-electrode cell comprising an Ag/AgCl in 3 M KCl as the reference electrode, a platinum rod as the counter electrode, and a glassy carbon electrode (GCE) coated with the investigated sample as the working electrode. The GCE was coated as follows: 10 mg of the sample was homogeneously dispersed in 0.95 mL of ethanol and 0.05 mL of 5 wt.% Nafion using an ultrasonic bath. Then, 10 μL of the suspension was placed on the surface of the GCE (0.071 cm^2^). A uniformly distributed sample was obtained on the GCE surface after solvent evaporation. Characterization of the samples was performed in 1 mM ferro/ferricyanide solution in 0.2 M KCl by cyclic voltammetry (CV) and electrochemical impedance spectroscopy (EIS). Mott–Schottky measurements were carried out at a frequency of 1000 Hz, with the voltage applied in successive steps of 50 mV from 1 V to −1 V. The measurements were performed in 0.1 M Na_2_SO_4_ (pH = 5.6).

The textural properties of the samples were determined from N_2_ adsorption–desorption isotherms obtained at −196 °C using a Sorptomatic 1990 Thermo Finnigan (Thermo Fisher Scientific, Waltham, MA, USA) apparatus. Before measurement, samples were degassed at the preparation site for 4 h at room temperature under vacuum, and then for a further 24 h at 80 °C under a vacuum pressure better than 0.3 Pa. The N_2_ isotherms were analyzed using ADP 5.13 Thermo Electron Software. The specific surface areas (*SSA*_BET_) of the samples and BET constant (C_BET_) were calculated using the Brunauer–Emmett–Teller (BET) equation [15] from the part of the adsorption isotherms selected according to the Rouquerol criteria [16]. The mesopore volume (V_meso_) was obtained from the desorption branch of the isotherm for the p/p0 region corresponding to the mesopores.

## 3. Results and Discussion

The XRD pattern of graphitic carbon nitride (GCN) is typically characterized by two main peaks: one centered around 13°, originating from intralayer ordering of tri-s-triazine building units, and another around 27.5°, attributed to the distance between layers interconnected by weak van der Waals forces [6].

**Figure 1:**
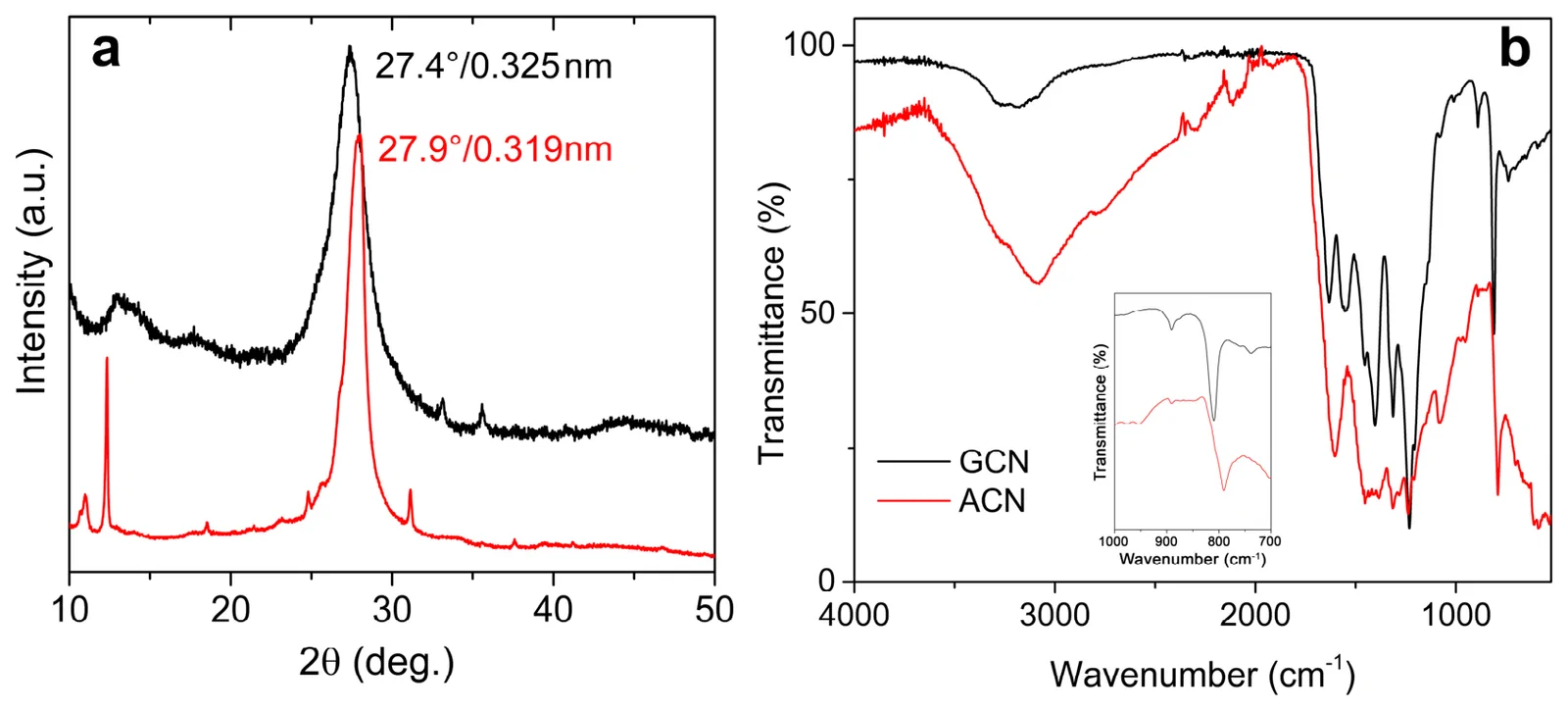
(**a**) XRD pattern and (**b**) FTIR spectra of GCN (black line) and ACN (red line). Inset: enlarged FTIR spectra in the 1000–700 cm^−1^ region, showing a shift in the characteristic heptazine band towards lower wavenumber values.

The XRD pattern of ACN (Figure 1a) provided the first indication that severe disruption of the ordered layered structure of GCN occurred under the applied chemical treatment, which was unexpected, as no similar disruption has been reported in previous studies using the same or similar chemical treatment of carbon nitride [3,14].

The peak at 12.9°, associated with the intralayer structure linked by hydrogen bonding, is absent in the XRD pattern of the ACN sample. The complete disappearance of this peak indicates that long-range order is severely destroyed due to cleavage of hydrogen bonds between heptazine-based melon chains. As hydrogen bonds in the GCN structure are formed between residual amino and imino groups, this strongly suggests that the applied experimental procedure led to the removal of these bridging groups. [4] Additionally, a shift in the interlayer-related peak from 27.4° to 27.9, indicates delamination of the material, resulting in fewer, more densely stacked layers, as the d-spacing was reduced from the initial 0.325 nm (GCN) to 0.319 nm (ACN).

**Figure 2:**
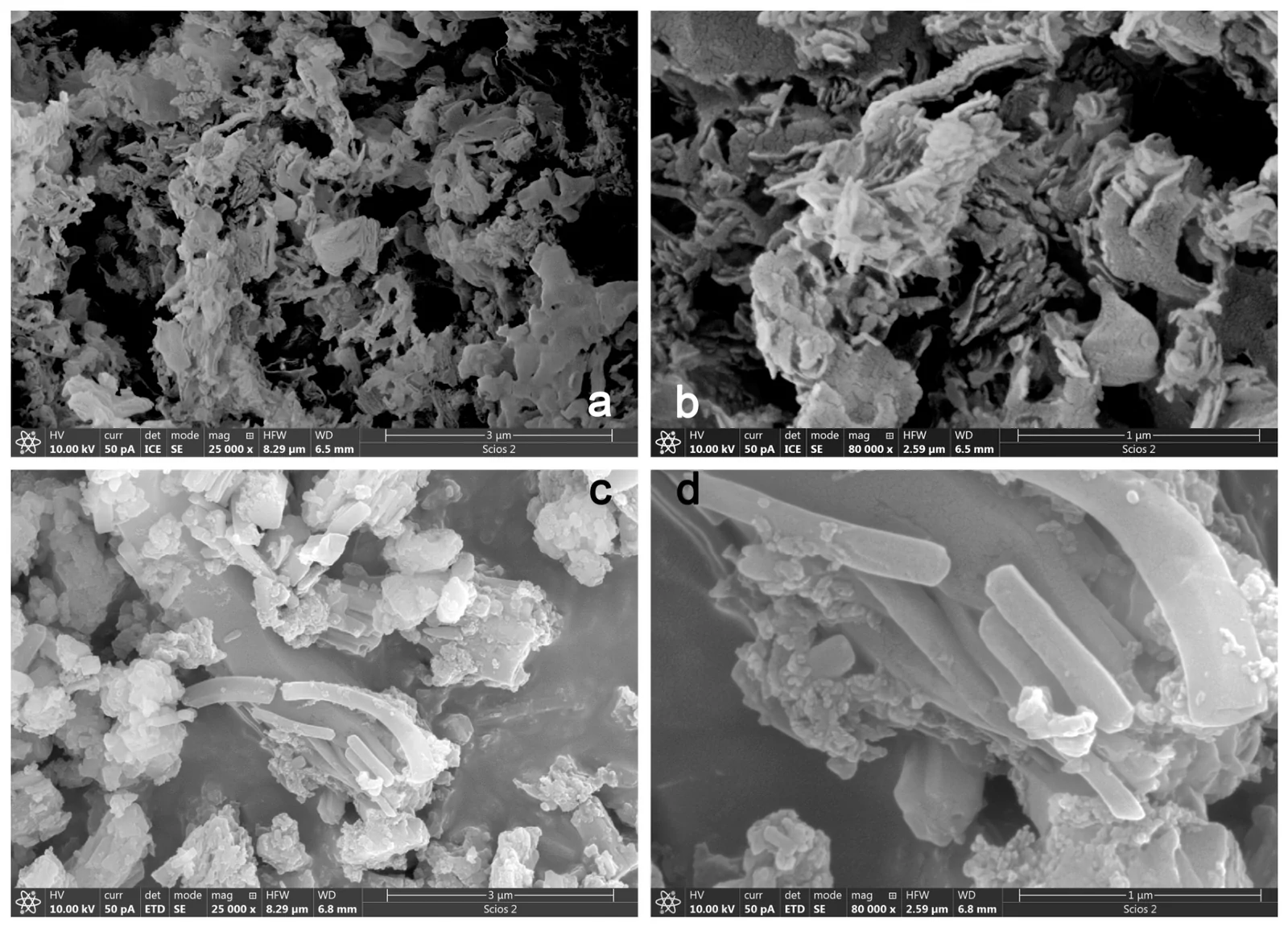
SEM images of (**a**,**b**) GCN and (**c**,**d**) ACN.

In addition to the notable changes in the two carbon nitride-related peaks, the XRD pattern of the ACN sample is characterized by the presence of less intense but very sharp peaks at 10.9, 12.3, 18.5, 23.1, 24.8, and 31.1°. These peaks can be ascribed to melem hydrate, based on comparison with the experimental and calculated diffraction patterns reported by Dai et al. [17] in Figure 2. In particular, the positions and distribution of the sharp peaks observed in the ACN sample show good agreement with the diffraction pattern of rod-shaped melem hydrate (Mhr), whose trigonal crystal structure (R$\bar{3}$c) was established in the study by Dai et al. [17]. This correspondence across several reflections supports the identification of Mhr as a crystalline component of the chemically treated material, while the broader diffraction features indicate the coexistence of less ordered carbon nitride-based structures. According to the literature, pristine MH has so far been synthesized by only a few research groups using a bottom-up approach [17,18], but it is also reported as melem or melem hydrate obtained when C_3_N_4_ or C_3_N_5_ is treated with concentrated H_2_SO_4_ or a mixture of concentrated H_2_SO_4_ and HNO_3_ [8,9,11]. Melem hydrate units are usually composed of six melem molecules interconnected by hydrogen bonds, forming hexagonal porous structures, as initially described by Makowski [19]. Due to such an arrangement, a channel filled with water molecules is formed in the center of the structure.

Depending on the synthetic procedure and calcination temperature, melem hydrate can form rod- or parallelogram-shaped microcrystals [17,18]. Both the resulting morphology and the amount of water present in the structure significantly affect the position of the peak around 12°. The XRD pattern of rod-shaped melem hydrate is characterized by a peak at 12.3°, while for the parallelogram-shaped MH, this peak is shifted to lower 2θ angles.

Structural changes induced by chemical treatment are confirmed by comparing the FTIR spectra of the parent and treated samples (Figure 1b). As previously stated [6], the heptazine (tri-s-triazine)-based structure of GCN is characterized by bands in the 1200–1650 cm^−1^ region, originating from stretching vibrations of C–N bonds in heterocycles. Their number and intensity correlate with the crystallinity of the sample, i.e., the degree of condensation. Additionally, the presence of side amino groups is confirmed by a weak, broad band centered at about 3250 cm^−1^ and a sharp band at 890 cm^−1^. The GCN heptazine-based structure is further evidenced by the out-of-plane bending vibration of the ring structure with a band at 815 cm^−1^. After aggressive chemical treatment, the FTIR spectrum of ACN reveals a profound structural transformation, indicating severe acid-catalyzed depolymerization of the polymeric framework. The fine, well-resolved structure of the C–N and C=N bands between 1200 and 1650 cm^−1^ is lost and replaced by a broad, dominant band. While the absence of sharp, well-defined melem monomer doublets (typically at 1622 and 1471 cm^−1^ [9]) might initially suggest incomplete depolymerization, this spectral broadening is highly characteristic of the proposed formation of melem hydrate. In such a structure, the extensive network of strong intermolecular hydrogen bonds between the newly formed melem molecules and intercalated water significantly alters the vibrational environment, merging these stretching modes into a single broad band around 1600 cm^−1^. This is strongly supported by the appearance of a massive, extremely broad absorption band dominating the high-frequency region (centered at ~3100 cm^−1^), which clearly corresponds to a large amount of heavily hydrogen-bonded –OH and –NH groups [11].

The most definitive evidence for the destruction of the polymeric network is the pronounced shift in the characteristic heptazine ring breathing vibration. The sharp band at 815 cm^−1^ in GCN almost completely disappears, shifting to approximately 790 cm^−1^ in ACN (inset in Figure 1b). This shift is a well-established marker for the transition from a condensed polymeric melon/heptazine network to isolated melem (2,5,8-triamino-heptazine) molecules [9].

Together with the changes observed in the XRD pattern, the FTIR results indicate that the chemical treatment induced extensive structural transformation of GCN, including fragmentation of the original polymeric structure and the formation of melem-based species.

Structural changes are evident in the evolution of the layered and elongated morphology of the ACN sample, as observed in SEM images (Figure 2). While GCN consists of stacked layers, ACN comprises smaller, layered fragments with a very broad size distribution, as well as rod-shaped features up to a few micrometers in size, whose morphology is consistent with the rod-shaped melem hydrate crystals reported in the available literature [17,18]. The high inhomogeneity of shapes and sizes can be attributed to the relatively short duration of the chemical treatment, but SEM alone does not provide definite conclusions about the formation mechanism of melem hydrate-based structures. Exclusively MH hexagonal or parallelogram rods are characteristic of the bottom-up approach, whereas in the top-down synthesis approach used in this study, multiple steps in the formation of melem hydrate-based structures clearly overlapped. It can be speculated that the acidic mixture initially caused delamination of the GCN stacked layers, forming thinner structures, which were later fragmented into smaller heptazine-based units. These fragments were likely subsequently self-assembled into melem hydrate-based rods, possibly not only during the synthetic procedure, but, considering the size of the stacked rod superstructures, even during the initial phases of the post-treatment dialysis step. However, even the rods are not uniformly structured, which, based on conclusions drawn by Kanai et al. [18], may be a consequence of the amount of water added to the reaction system. According to their findings, the formation of a mixture of plate-shaped microcrystals together with hexagonal rods occurred with increasing water content, up to 40%. As the ratio of water in our synthesis and subsequent treatment was much higher, together with overlapping steps of fragmentation, melem formation, and rod assembly, the resulting inhomogeneity of shapes and sizes and the simultaneous presence of trigonal, pentagonal, and hexagonal melem hydrate-based rods are not unexpected.

**Figure 3:**
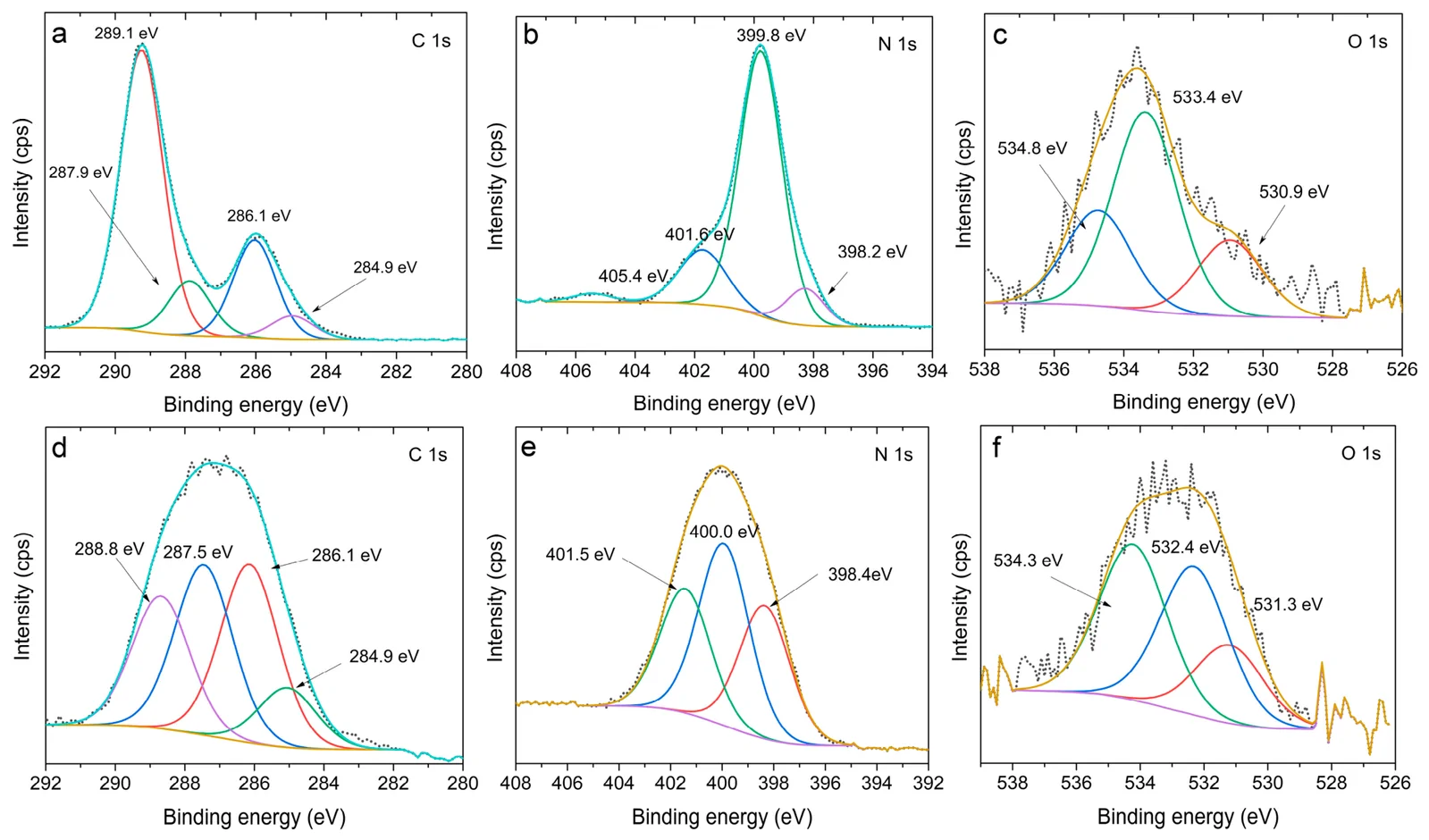
High-resolution XPS spectra of core levels for (**a**–**c**) GCN and (**d**–**f**) ACN.

To estimate changes in surface chemistry, XPS spectra of both samples were recorded (Figure 3 and survey spectra in Figure S1). The deconvoluted C 1s spectrum of the GCN sample exhibited four components (Figure 3a): adventitious carbon at 284.9 eV, C–N bonding at 286.1 eV, an N–C=N bond (carbon from the aromatic ring) at 287.9 eV and, the most prominent peak at 289.1 eV, which is usually assigned to C=O moieties, i.e., the carboxyl group [7,8,10,20,21], formed as a result of partial oxidation of the surface. However, as the estimated percentage of oxygen in the GCN sample is only about 3% (Figure S1), assigning this peak exclusively to a C=O moiety is not chemically realistic, especially considering the absence of oxygen-related bands in the FTIR spectrum of this sample. According to some authors [8,11,22,23], the peak at 289.1 eV originates from sp^2^- hybridized carbon in the aromatic ring connected to an amino group (C–NH_2_). Such results are expected for urea-derived GCN, as this material is characterized by the presence of pores and imperfections in the layers, which are actually terminations of the heptazine-based structure, rich in amino groups [6]. Comparison of the peak areas revealed that the contribution of the C–NH_2_ peak relative to the N–C=N peak is about three times higher, further emphasizing the porosity of urea-based GCN. This ratio of the corresponding peaks in the ACN C 1s spectrum (Figure 3d) is as low as 0.85, indicating significant changes in surface chemistry after the applied chemical treatment.

At the same time, the N 1s spectrum (Figure 3b) of GCN can be deconvoluted into four bands, with the contribution of the N-O bond at 405.4 eV being almost negligible. The main contribution arises from the peak with a maximum at 399.8 eV, assigned to N_3_C three-coordinated structural nitrogen, i.e., the N-[C]_3_ bond, corresponding to nitrogen atoms connecting s-triazine rings [24] that form the heptazine backbone of graphitic carbon nitride. The N-[C]_3_ peak area, compared to the C-NH_2_ peak [5] area (maximum at 401.6 eV) and the C-N=C peak [5] area (N_2_C two-coordinated structural nitrogen, maximum at 398.2 eV), is 0.73:0.21:0.057. Such a low proportion of N_2_C suggests incomplete condensation or aromatic ring opening in GCN, consistent with previous findings.

However, after the applied chemical treatment (Figure 3e) this ratio changes substantially to 0.43:0.34:0.25, i.e., the relative contribution of the N-[C]_3_ peak decreases, consistent with the observed fragmentation.

High-resolution O 1s spectra of GCN and ACN are presented in Figure 3c and Figure 3f, respectively. As stated above, the oxygen contribution in both samples is very low, resulting in a very weak line with a high noise-to-signal ratio. In the deconvoluted O 1s spectra of both samples, three major lines are identified: at 530.9–531.3 eV, at 533.4–532.4 eV, and at 534.8–534.3 eV. Assignment of these lines is challenging, as literature data are inconsistent. The component at about 533 eV can be assigned to a C=O bond [13,25]. However, as the presence of carboxyl oxygen is not supported by the C 1s or FTIR spectra, it can be assumed that this contribution more likely originates from surface-adsorbed water [26,27]. Nonetheless, any further discussion would be speculative, and it can only be concluded that the applied chemical treatment did not result in significant incorporation of oxygen into the carbon nitride matrix.

Investigation of the textural properties of the samples showed that structural changes, induced by the applied chemical treatment, led to a slight decrease in the specific surface area (*SSA*_BET_), as indicated in Table 1. Additionally, the BET constant value decreased, indicating a reduction in adsorbent–adsorbate interaction.

**Figure 4:**
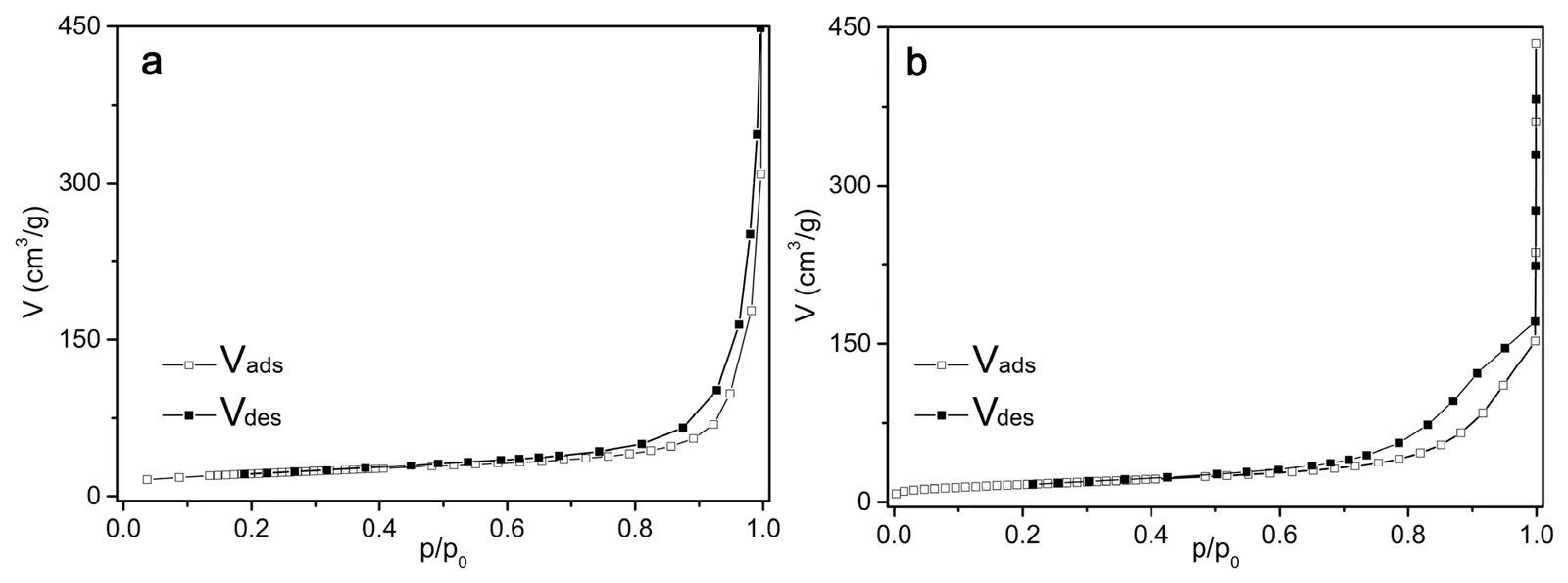
N_2_ adsorption/desorption isotherms of (**a**) GCN and (**b**) ACN.

The N_2_ isotherms of the GCN sample (Figure 4a) correspond to Type II isotherms, according to IUPAC nomenclature [28], characteristic of non-porous or macroporous materials, with no characteristic inflection point related to the completion of monolayer coverage. Instead, the shape of the isotherm resembles an overlap of monolayer and multilayer coverage steps. The isotherms are characterized by a very narrow hysteresis, which can be identified as a Type H3 loop, common for non-rigid aggregates of plate-like particles [28].

The structural changes induced by the applied chemical treatment are reflected in the N_2_ isotherms of the ACN sample (Figure 4b). Although the isotherm exhibits a similar Type II shape, the hysteresis loop is more pronounced, indicating changes in pore shape and/or connectivity of the mesopores.

Comparison of the content of mesoporous regions and their distribution calculated by the BJH method [29] (Figure S2, Supplementary Materials) shows that the applied chemical treatment altered the pore-size distribution, although the cumulative mesopore volume remained almost unchanged. The GCN sample exhibits a broad mesopore size distribution with a median pore diameter of approximately 25 nm. In contrast, the ACN sample displays a more uniform mesopore size distribution, with a lower median pore diameter of about 14.1 nm and a dominant pore size of around 11 nm.

An additional distinctive feature of the isotherm is the pronounced steep increase, almost parallel to the *y*-axis, near p/p_0_ → 1, indicating unrestricted multilayer adsorption on the available external surfaces of the particles. As the condensed liquid layer on the particle surfaces grows infinitely thick, it leads to capillary condensation within the macroscopic interparticle voids. This behavior strongly suggests that the ACN sample consists of small, highly dispersed entities with available external surface area, creating a highly open, loose network capable of significant macroscopic void filling.

**Figure 5:**
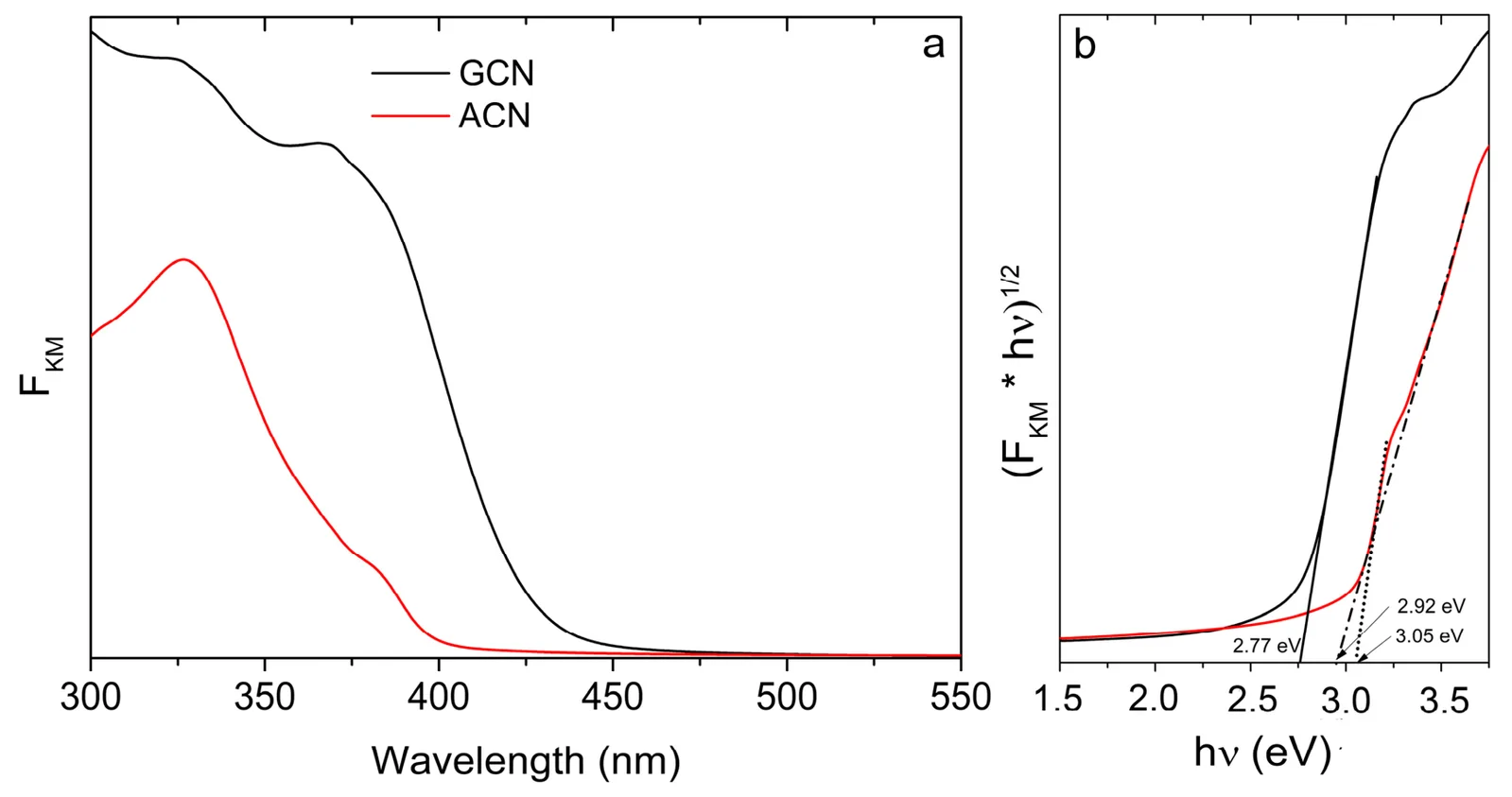
(**a**) Kubelka–Munk transformed UV-vis DR spectra of GCN (black line) and ACN (red line) and (**b**) Tauc plots of GCN (black line) and ACN (red line).

To assess the optical characteristics of the synthesized samples, UV-vis DR spectra were obtained. In Figure 5a, the transformed Kubelka–Munk spectra are presented. A broad maximum at approximately 370 nm in the GCN spectrum is typical of π-π* transitions in conjugated systems [5,30]. However, the same maximum in the ACN spectrum is barely visible, which is in accordance with the disruption of the π-conjugated network, as previously stated. Moreover, the absorption edge is blue-shifted, consistent with the whitish color of the sample. The obtained Tauc plots (Figure 5b) confirmed a shift in the band gap energy toward higher values. The Tauc plot of the ACN sample is characterized by two linear regions: one extrapolated to an energy of 2.92 eV and the other to approximately 3.05 eV. Both values are higher than the estimated band gap energy of the parent GCN material, which is about 2.77 eV.

**Figure 6:**
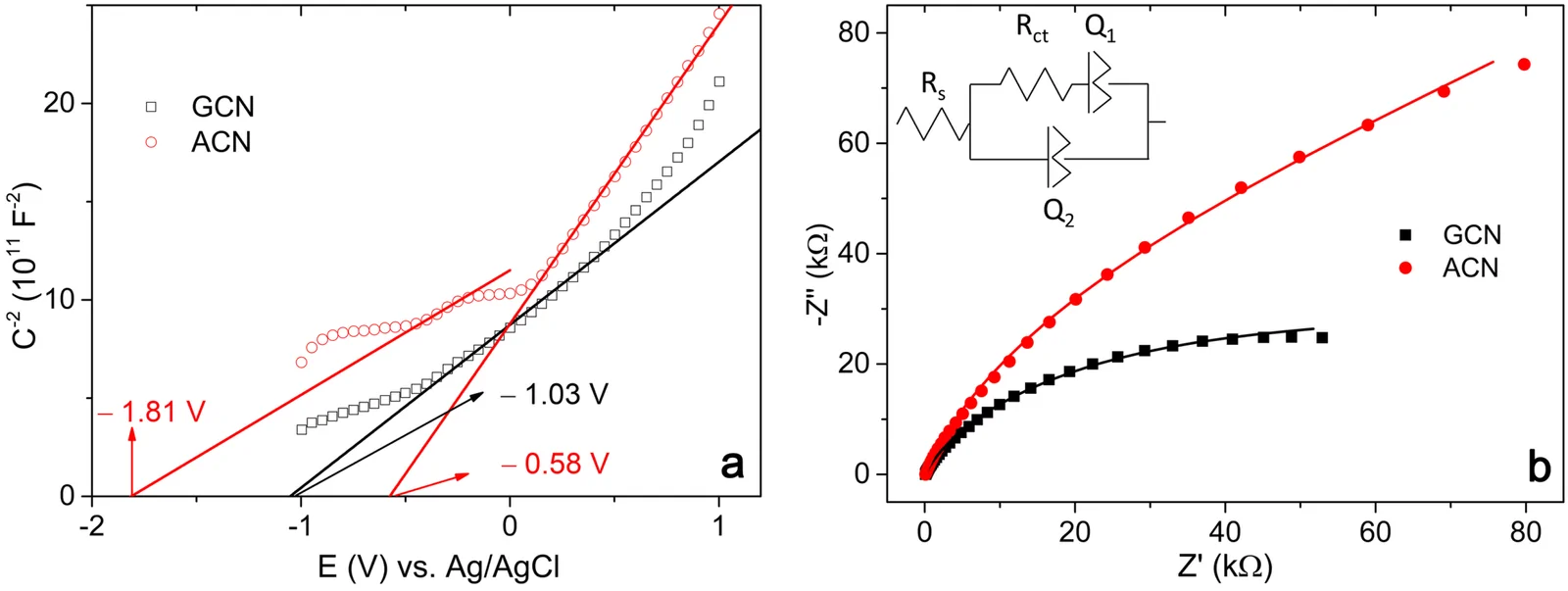
(**a**) Mott–Schottky plots of GCN (black) and ACN (red) measured at 1000 Hz in 0.1 M Na_2_SO_4_ solution (pH = 5.6) and (**b**) Nyquist plots of GCN (black) and ACN (red). Inset: Equivalent electric circuit used to fit EIS data.

To estimate the effect of chemical treatment on electronic energy levels, Mott–Schottky plots (Figure 6a) were recorded, and characteristic potentials were determined (Table 2). Both samples exhibit curves with positive slopes, indicating n-type semiconductor behavior [31]. For n-type semiconductors, the conduction band edge potential is generally considered to be 0.1 [30] to 0.3 V [32] more negative than the flat-band potential. Assuming a difference of 0.1 V, the conduction band edge of GCN would be at −0.92 V (vs. SHE). As the band gap of this material is estimated to be 2.77 eV, the valence band edge potential would be at +1.85 V (vs. SHE).

In contrast, the Mott–Schottky plot of the ACN sample exhibited two slopes, extrapolated to values of −0.37 V and −1.60 V (vs. SHE), indicating a significantly different electronic structure compared to the parent GCN. However, before assigning these values to the flat-band potentials of the ACN mesh components, two considerations must be addressed:

• The fragmentation of the layered GCN structure inevitably leads to the creation of defect states on the material’s surface. These defects can be donor-type, such as nitrogen vacancies and cyano groups, or acceptor-type, such as carbon vacancies or oxygen-containing groups (-OH, -C=O, or -COOH). Such surface states can create high-density energy states within the bandgap, effectively capturing charges and pinning the Fermi level (charge-neutral level) to a fixed potential value [33,34]. Consequently, potentials obtained by Mott–Schottky measurements show lower (more positive) values at the intercept [35], which is a possible explanation for the estimated potential of −0.37 V.
• Chemically induced fragmentation reduces the degree of polymerization, thereby shortening the conjugated system relative to the parent GCN. Consequently, electrons are less delocalized; that is, fragmentation leads to improved electron confinement, and a negative shift in the flat-band potential could be expected [36,37,38,39] compared to the flat-band potential of the parent GCN. However, the estimated potential of −1.60 V would imply a significant negative shift of almost 0.8 V, placing the conduction band minimum at approximately −1.7 V, which would be characteristic of material capable of rapid and intense formation of superoxide radical anions. It would also mean that, once illuminated, it would probably be susceptible to photocorrosion.

To clarify the origin of the estimated potentials and probe interfacial processes [40], further characterization was performed using electrochemical impedance spectroscopy, and the resulting Nyquist plots are shown in Figure 6b. The modified Randles circuit (inset in Figure 6b) was used to fit the spectra. Parameters obtained from fitting the Nyquist plots are presented in Table S1 (Supplementary Materials). The results showed that the acid-treated sample had a higher charge transfer resistance (58 kΩ for GCN vs. 83 kΩ for ACN), consistent with altered charge transfer behavior following fragmentation.

Considering these results, the estimated potential at −1.60 V vs. SHE should be treated as an apparent value rather than a clear band-edge position. The value of this potential is determined by the defect states in the fragmented phase, which act as electron-trap states. Further confirmation can be found by comparing Mott–Schottky plots measured at higher frequency (Supplementary Materials, Figure S3), where the higher potential remains at a value of −0.37 V vs. SHE, while the feature correlated with defect states disappears. The acid treatment altered the surface charge state [41], resulting in the apparent potential value (−1.60 V), while the true band-edge position (−0.37 V) remained stable.

**Figure 7:**
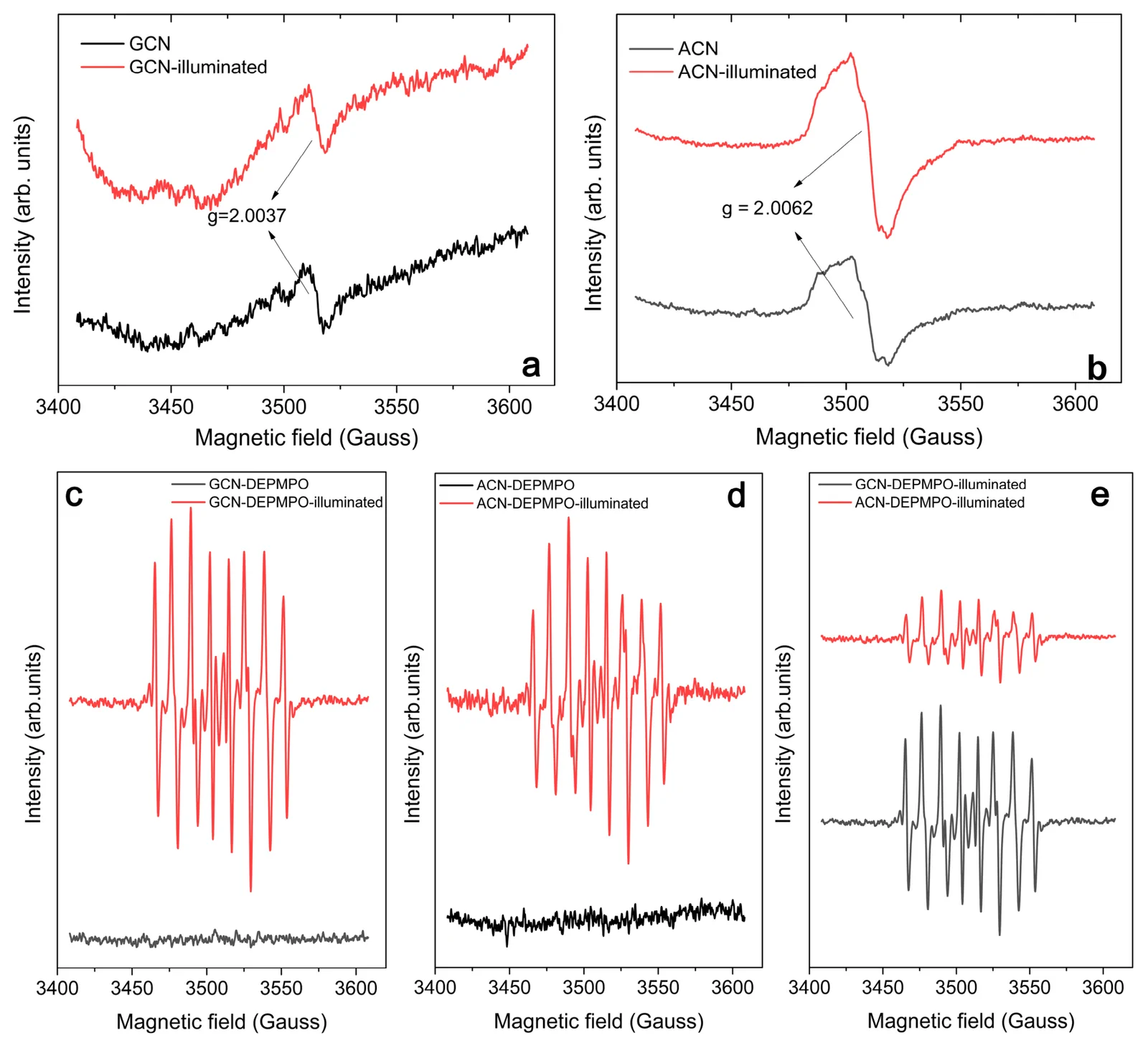
EPR spectra of (**a**) GCN and (**b**) ACN as dry powders, (**c**) GCN and (**d**) ACN in aqueous suspension with the DEPMPO spin-trap, and (**e**) comparison of the DEPMPO/OOH adduct signals obtained after 10 min of visible light irradiation of GCN (black) and ACN (red).

EPR measurements were conducted to further verify the origin of the recorded potentials. In Figure 7a, the EPR spectrum of the GCN sample shows a weak Lorentzian line, originating from unpaired electrons in the hybridized aromatic system [42]. After illumination (red line, Figure 7a), the shape, intensity, and g-value remain unchanged, indicating that photogenerated charges do not contribute to the signal, probably due to the fast charge carrier recombination characteristic of GCN. However, the EPR spectrum of ACN (Figure 7b, black line) shows a more intense peak with characteristic splitting, which may indicate different electronic and structural environments, consistent with the coexistence of different phases in the material. Upon illumination (Figure 7b, red line), the peak area increases by a factor of 1.68, suggesting possible trapping of photogenerated charges in shallow trap states. Given the proven fragmentation of the polymeric structure, shallow trapping of charges could occur at under-coordinated N atoms and/or at heptazine edge terminal structural defects [42].

To evaluate the effect of the applied chemical treatment on the formation of short-lived radicals under visible light irradiation, the EPR spin-trapping technique using DEPMPO as a spin-trap was employed. Both GCN and ACN samples (Figure 7c,d) showed no detectable signal in the dark, indicating that neither material generates radicals without visible light. Upon illumination, a strong EPR signal characteristic of the DEPMPO-superoxide adduct (DEPMPO/OOH) was observed in both samples. Notably, the DEPMPO/OOH adduct signal for GCN was approximately 2.5 times higher than that for ACN (Figure 7e), suggesting that a fraction of the photogenerated charges may remain trapped in defect states.

This ratio was estimated from the heights of several characteristic peaks of the experimental spectra and should therefore be considered semi-quantitative. Besides charge trapping, other factors may contribute to this difference. Owing to its blue-shifted absorption edge and suppressed π–π* absorption (Figure 5), ACN absorbs less visible light than GCN, and light scattering by the micrometer-sized melem hydrate rods may further reduce its effective absorption. In addition, the specific surface area of ACN is about 21% lower than that of GCN (Table 1), although normalization per unit surface area still leaves an approximately two-fold difference. Differences in sample dispersion are unlikely to account for the lower signal, as the more hydrophilic ACN is expected to disperse at least as readily as GCN. Restricted spin-trap accessibility is also unlikely, since DEPMPO (below 1 nm) is much smaller than the mesopores of both samples. Nevertheless, the remaining difference is consistent with charge trapping, as supported by the light-induced increase in the EPR signal of dry ACN powder (Figure 7b), the higher charge transfer resistance of ACN, and the Mott–Schottky results.

The results obtained in this study suggest that the widely cited chemical resistance of graphitic carbon nitride should be regarded with caution, as it strongly depends on the structure of the starting GCN. As shown previously [6], urea-derived GCN possesses a highly porous structure originating from gas release during synthesis. Such a porous system is particularly susceptible to reactions with strong acids such as H_2_SO_4_ and HNO_3_. Due to the strong oxidizing ability and direct cleavage function of SO_4_^2−^ and NO^3−^ [5,8,10,11], not only delamination but also fragmentation of the GCN layers occurred, potentially involving cleavage of C–N bonds connecting heptazine-based units and the release of melem species. The subsequent addition of H_2_O_2_ likely further accelerated this process, possibly involving in situ formation of Caro’s acid with H_2_SO_4_, promoting further release of melem, although its formation and contribution were not directly investigated. Upon addition of water and during dialysis, the released melem species may have undergone self-assembly, resulting in the observed rod-like melem hydrate-based structures.

Although almost the same treatment of graphitic carbon nitride is reported by other authors [3,13], no formation of MH or a fragmented phase is reported. This discrepancy can be attributed to the use of different carbon nitride precursors (melamine/dicyandiamide), resulting in a more compact layered structure than that of urea-derived GCN, and consequently greater resistance to the oxidative action of acids. In the case of urea-derived GCN, the results show that the applied oxidative treatment reconstructs the carbon nitride surface into a defect-dominated interface that controls charge transfer processes rather than only changing surface functions. Additionally, fragmentation of the system starts quickly, providing melem units for the self-assembly of MH rods. However, adjustment of reaction conditions is required to obtain a more homogeneous and uniform MH system.

## 4. Conclusions

Urea-derived GCN was chemically treated with a mixture of H_2_SO_4_/HNO_3_/H_2_O_2_, then dialyzed against water. XRD results indicated the formation of melem hydrate (MH), while SEM revealed tri-, penta-, and hexagonal rod-like morphologies consistent with melem hydrate-based structures.

FTIR analysis further supported the destruction of the heptazine-based backbone, as the characteristic out-of-plane vibration at approximately 815 cm^−1^ disappeared and a new band at around 790 cm^−1^ emerged, consistent with melem-type units. Extrapolation of two linear regions of the Tauc plot of the ACN sample yielded energies of 2.92 and 3.05 eV. Therefore, the ACN sample should be considered a mesh of MH and defect-rich melem-based fragments, where the fragmented phase significantly affects the overall electronic properties. Specifically, Mott–Schottky and EPR measurements suggested the presence of electron-trapping defect states, which, together with the reduced visible-light absorption, may limit charge carrier availability and reduce the capacity of the material for photogenerated electron transfer to oxygen.

The presented results show that acid treatment of urea-based g-C_3_N_4_ provides a viable low-temperature route to melem hydrate-enriched structures.

## Figures and Tables

**Table 1:** Textural properties of GCN and ACN samples determined by N_2_ sorption measurements.

Sample	*SSA*_BET_, (m^2^/g)	*C_BET_*	*V*_mes,_ (cm^3^/g)	*D*_mes,_ (nm)
GCN	75	114	0.234	24.7
ACN	59	74	0.232	14.1

**Table 2:** Characteristic potentials derived from Mott–Schottky analysis.

Sample	E_MS_ (V) vs. Ag/AgCl	E_MS_ (V) vs. RHE	E_MS_ (V) vs. SHE
GCN	−1.03	−0.49	−0.82
ACN	−0.58	−0.04	−0.37
	−1.81	−1.27	−1.60

## Data Availability

The original contributions presented in the study are included in the article/Supplementary Materials; further inquiries can be directed to the corresponding authors.

## Supplementary Materials

The following supporting information can be downloaded at: https://www.mdpi.com/article/10.3390/polym18212664/s1, Figure S1: XPS survey spectra of GCN and ACN; Figure S2: Mesopore size distribution estimated by the Barrett, Joyner and Halenda (BJH) method for (a) GCN and (b) ACN. TSE stands for tensile strength effect; Table S1: The parameters obtained by fitting Nyquist plot with the equivalent circuit. Figure S3: Mott-Schottky plots of ACN measured at 1000 Hz (red) and 2000 Hz (green) in 0.1 M Na_2_SO_4_ solution (pH = 5.6).
