# Supplementary material for: Urea-Derived Carbon Nitride Fragmentation: A Route to Melem-Based Superstructures
Source: Polymers (Basel). 2026 Oct 28;18(21):2664. doi: 10.3390/polym18212664 (PMC13766023; doi:10.3390/polym18212664)
Supplement: Supplementary file 1 [file polymers-18-02664-s001.zip › polymers-4625364-Supplementary Materials.pdf]

polymers

MDPI

Supplementary Materials

Urea-Derived Carbon Nitride Fragmentation: A Route to Melem-Based Superstructures

Milica V. Carević [1,]*, Nadica D. Abazović [1,]*, Zorica D. Mojović [2], Jugoslav B. Krstić [2], Miloš D. Mojović [3], Tatjana D. Vulić [1] and Mirjana I. Čomor [1]

[1] Vinča Institute of Nuclear Sciences, National Institute of the Republic of Serbia, University of Belgrade, Mike Petrovića Alasa 12–14, Vinča, 11351 Belgrade, Serbia; tanja030@vin.bg.ac.rs (T.D.V.); mirjanac@vin.bg.ac.rs (M.I.Č.)

[2] University of Belgrade, Institute of Chemistry, Technology and Metallurgy, Department of Catalysis and Chemical Engineering,Njegoševa 12, 11000 Belgrade, Serbia; zorica.mojovic@ihtm.bg.ac.rs (Z.D.M.); jugoslav.krstic@ihtm.bg.ac.rs (J.B.K.)

[3] Center for Physical Chemistry of Biological Systems, BioScope Labs, Faculty of Physical Chemistry, University of Belgrade, Studentski trg 12–16, 11158 Belgrade, Serbia; milos@ffh.bg.ac.rs

* Correspondence: milicab@vin.bg.ac.rs (M.V.C.); kiki@vin.bg.ac.rs (N.D.A.)

XPS Measurements

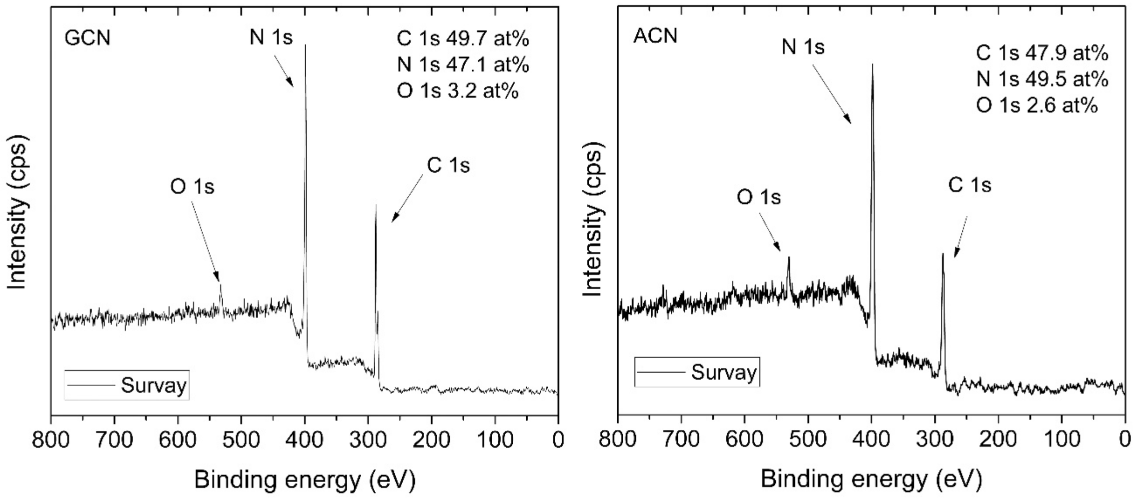

Figure S1. XPS survey spectra of GCN and ACN.

Textural Properties

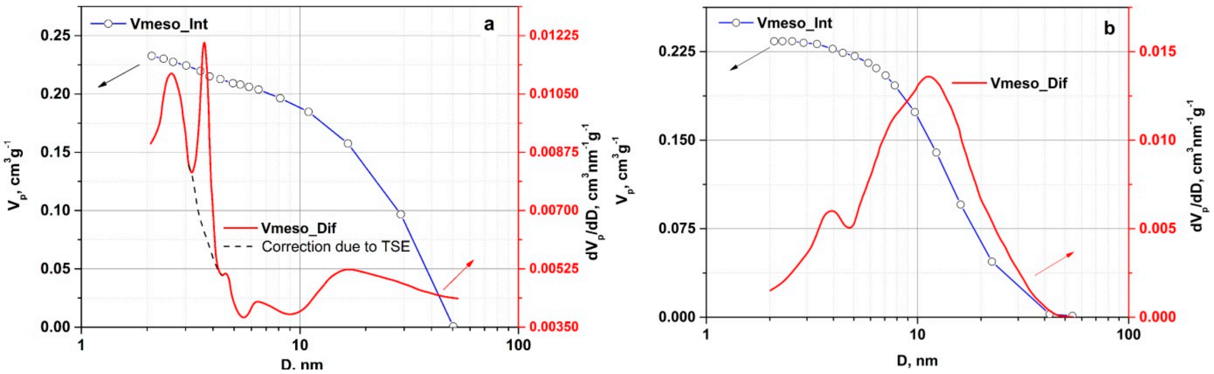

Figure S2. Mesopore size distribution estimated by the Barrett, Joyner and Halenda (BJH) method for (**a**) GCN and (**b**) ACN. TSE stands for *tensile strength effect.*

Electrochemical Measurements

The modified Randles equivalent circuit (inset in Figure 6b) composed of the internal resistance (R_s) in series with the parallel combination of the double-layer capacitance (Q_2) and the impedance of a faradaic reaction (serial combination of charge transfer resistance (R_{ct}) and diffusion (Q_1)) was used to fit the obtained spectra. The constant phase element was used rather than capacitance element to accommodate non-ideal capacitor behavior. The impedance of CPE is given as:

$$Z = \frac{1}{Y} = \frac{1}{(i\omega)^n Q}$$

where Y is CPE admittance, i is the imaginary unit, ω is the angular frequency, n is CPE exponent which is associated with the system inhomogeneity and Q has the numerical value of the admittance (1/ |Z|) at ω = 1 rad/s.

Table S1. The parameters obtained by fitting Nyquist plot with the equivalent circuit.

**Electrode**	**R_s (Ω)**	**R_{ct} (kΩ)**	**Y_1 (μSs^{n1})**	**n1**	**Y_2 (μSs^{n2})**	**n2**	**χ^2**
GCN	99	58	69	0.6	7.4	0.8	0.106
ACN	103	83	12	0.4	6.8	0.8	0.045

*χ^2 parameter that shows goodness of the fit.

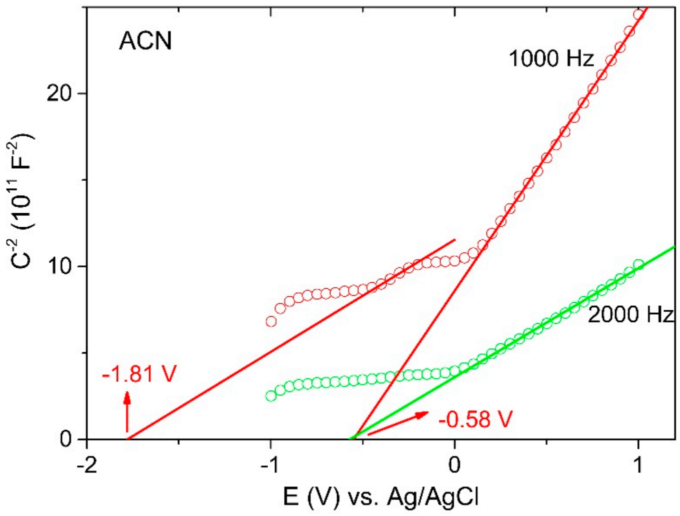

Figure S3. Mott-Schottky plots of ACN measured at 1000 Hz (red) and 2000 Hz (green) in 0.1 M Na_2SO_4 solution (pH = 5.6).
